# Improving the surveillance and response system to achieve and maintain malaria elimination: a retrospective analysis in Jiangsu Province, China

**DOI:** 10.1186/s40249-022-00939-3

**Published:** 2022-02-21

**Authors:** Yuanyuan Cao, Guangyu Lu, Chris Cotter, Weiming Wang, Mengmeng Yang, Yaobao Liu, Cheng Liang, Huayun Zhou, Yan Lu, Jun Yan, Guoding Zhu, Jun Cao

**Affiliations:** 1 Key Laboratory of National Health Commission (NHC) On Parasitic Disease Control and Prevention, Jiangsu Provincial Key Laboratory On Parasite and Vector Control Technology, Jiangsu Institute of Parasitic Diseases, Wuxi, 214064 Jiangsu China; 2 Institute of Public Health, Medical College of Yangzhou University, Jiangsu Province, Yangzhou city, 225000 China; 3 Malaria Elimination Initiative, Institute for Global Health Sciences, University of California San Francisco, San Francisco, CA USA; 4 Department of Women’s and Children’s Health, International Maternal and Child Health, Uppsala University, Uppsala, Sweden; 5 Center for Global Health, School of Public Health, Nanjing Medical University, Nanjing, 211166 China; 6 Health and Quarantine Department, Nanjing Customs, Nanjing, 210001 China; 7 Chinese Center for Disease Control and Prevention, Beijing, 102206 China

**Keywords:** China, 1-3-7 approach, Surveillance and response, Malaria, Elimination, Imported case, Jiangsu, Retrospective study

## Abstract

**Background:**

Following initiation of China’s National Malaria Elimination Action Plan (NMEAP) in 2010, the ‘1-3-7’ approach was developed and rolled out in China to facilitate the malaria elimination programme and accelerate malaria elimination. This study aims to summarize and condense these experiences through a retrospective analysis in Jiangsu Province, which could be adapted and applied in other malaria elimination settings worldwide.

**Methods:**

A retrospective analysis of imported malaria cases into China identified through an improved surveillance and response system in Jiangsu Province was carried out for the period of 2001–2020. To improve the malaria surveillance and response system, Centers for Diseases Control and Prevention from the prefectures and counties in Jiangsu province conducted population-level health education to improve healthcare seeking behavior, strengthened capacity of health facilities to improve performance of malaria diagnosis and treatment, and raised the capacity of public health providers to improve implementation of the ‘1-3-7’ approach. Categorical variables were carried out by Chi square tests with *Fisher’s* exact correction.

**Results:**

From 2001 to 2020, a total of 9,879 malaria cases were reported in Jiangsu Province. Since 2012, no indigenous malaria cases have been reported in Jiangsu Province. However, in recent years, there has been a substantial increase of imported falciparum malaria cases. Between 2012 and 2020, an estimated 61.57 million individuals have benefited from population-level health education in Jiangsu Province. For healthcare-seeking services among the 2,423 imported malaria cases, 687 (28.4%) and 1,104 (45.6%) cases visited hospitals on the first day and the second day from symptom onset, respectively. A total of 1,502 (61.9%) cases were diagnosed on the first day at medical facilities. Jiangsu Province achieved 100%, 99.4% and 98.3% completion rate in terms of case detection and notification (within one day), case investigation (within three days) and foci response and disposition (within seven days), respectively. The improved surveillance and response system in Jiangsu Province plays an important role in preventing the re-introduction of malaria and maintaining the malaria-free status.

**Conclusions:**

Jiangsu Province has maintained its malaria-free status since 2012. The continuous improvement of a surveillance and response system plays an important role in the early detection and rapid response of potential malaria-related outbreaks in Jiangsu, China, and has important lessons for other malaria eliminating settings. Remaining vigilant in the detection of imported malaria cases and maintaining an active surveillance and response system is critical to sustain the success of malaria elimination.

**Graphic Abstract:**

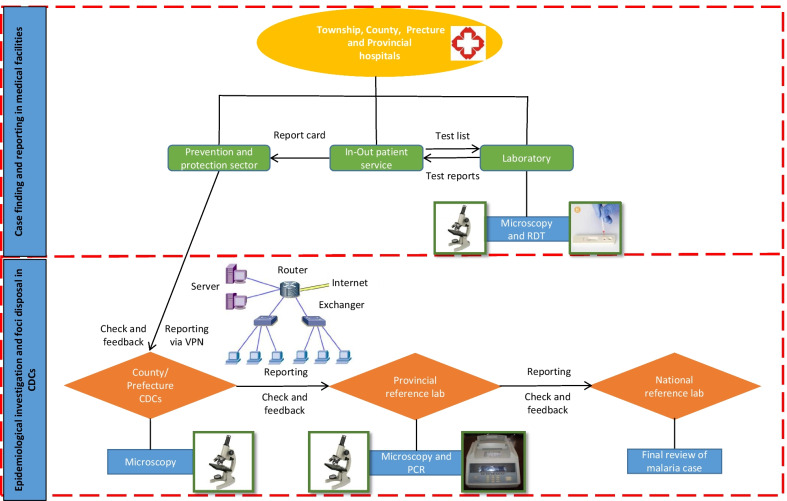

## Background

Malaria is a parasitic disease transmitted by mosquitoes that endangers people's health and safety across the world. In 2020, it was estimated that 247 million people were infected with malaria, including approximately 627,000 malaria-attributed deaths [1]. In China in the 1940s, more than 30 million people were infected with malaria annually impacting China’s social and economic development. With government engagement and leadership on malaria control and elimination more recently, malaria has been greatly reduced. In 2009, malaria cases decreased to 14,000 annually nationwide down from approximately 300,000 cases in 2001, a 95.3% reduction [2]. Building on their progress in controlling malaria, China launched its National Malaria Elimination Action Program (NMEAP) in 2010 with the goal of elimination by 2020 [3]. Since the launch of NMEAP, the malaria burden has further decreased. In 2017, locally acquired malaria transmission was effectively interrupted for the first time in China [4]. In June 2021, China was certified as malaria-free by the World Health Organization [5].

Jiangsu was a highly-endemic malaria province with an annual incidence rate of 24.9% during the 1960s. Through the implementation of comprehensive measures such as mosquito-borne source control, standard treatment and follow-up of malaria cases, and mass drug administration (MDA) [6], annual incidence of malaria declined to 0.1 per 10,000 population from 2008–2010 in Jiangsu Province. The number of indigenous malaria cases in Jiangsu Province dropped sharply from more than 1,000 in 2001 to 13 in 2011 [7]. In line with NMEAP, Jiangsu Province launched its provincial malaria elimination action plan program in 2010.

In China, during the transition from control to elimination between 2010 and 2020, indigenous malaria cases decreased sharply and characteristics of malaria cases changed. Due to a changing malaria epidemiology and the need to accelerate the national goal for malaria elimination, the surveillance and response system needed to be adapted and strengthened to increase the capacity to detect and investigate each malaria case promptly. Specifically, China’s ‘1-3-7’ approach was first developed in Jiangsu Province and scaled up to a total 31 provincial-level administrative divisions (PLADs) in China in early 2012 [8, 9]. The ‘1-3-7’ approach includes case reporting within one day (1 day), case investigation within three days (3 days) and focus/foci investigation and action within seven days (7 days). This strategy is used as a core indicator to evaluate the quality of key malaria elimination activities for all PLADs in China. Global technical guidelines for malaria surveillance [10] have adopted the strategy, and is used as a template for other countries in the Greater Mekong Subregion [11].

To improve the ‘1-3-7’ approach for malaria surveillance and response, innovative interventions have been developed and implemented in Jiangsu Province to achieve and maintain the malaria elimination goal. This study aims to summarize and condense these experiences through a retrospective analysis of imported malaria cases into China, which could be adapted and applied in other malaria transmission settings worldwide.

## Methods

### Study site

Located in the southeast of China, Jiangsu Province covers an area of 102.6 thousand square kilometers accounting for 1.1% of the total area of China. Situated in a transition belt from a subtropical to temperate zone, Jiangsu Province has a typical monsoon climate. Annual rainfall in Jiangsu Province is 1000.4 mm, mainly concentrated from May to October. In Jiangsu Province, *Anopheles sinensis* and *An. anthropophagus* are the dominant mosquito vectors, and the climate and environment is suitable for the breeding of *Anopheles* mosquitoes from May to October when it coincides with malaria transmission season. [12]. There are a total of 13 prefectural cities and 100 counties (the administrative unit under prefecture) in Jiangsu Province. In the twentieth century, malaria has been widely prevalent in Jiangsu Province. After repeated struggles controlling malaria, through the use of standard control interventions such as mosquito-borne source control, standard treatment and follow-up of malaria cases, MDA, and implementing a case-based malaria surveillance and response, the Jiangsu Provincial malaria program brought malaria under control by 2016 [13]. In 2018, Jiangsu passed the provincial malaria elimination assessment organized by the National Health Commission (NHC), China [14].

### Data extraction

Aggregate data from all 13 prefectures of Jiangsu Province were collected from 2001 to 2020, including monthly and annual malaria paper-based reports and provincial malaria materials compiled for malaria prevention and control. In addition to the above mentioned, aggregate data on malaria cases in Jiangsu Province were extracted from 2005 to 2020 and downloaded from China’s routine diseases surveillance information system (CRDSIS), a real-time, web-based infectious diseases surveillance and response system. CRDSIS was established by China CDC in 2004 [15]. Basic case characteristics of the CRDSIS specific for malaria were collected from the database and include: indicators for the ‘1-3-7’ approach, malaria treatment, occupation, health facility admission date, time of symptom onset, and time of malaria case diagnosis.

### Data management and analysis

The datasets established included basic characteristics of all malaria cases and were double entered into Microsoft Excel (Microsoft Corp, Seattle, USA). The time elapsing (in days) was calculated for each malaria case episode by subtracting the date of symptom onset from the health facility admission date for all malaria cases in Jiangsu Province. Days between case diagnosis and initially seeking health care for all malaria cases were calculated. Categorical variables were carried out by Chi square tests with *Fisher’s* exact correction when the expected frequency in any cell was five or less. All statistical analyses were performed in STATA v.12 (Timberlake, College Station, TX, USA) and SPSS v.16.0 (Statistical Product and Service Solutions). Geographic information system (GIS)-based spatial analysis was conducted to demonstrate the malaria elimination assessment process at county level in Jiangsu Province. All spatial analyses were carried out by QGIS (Quantum GIS, v2.10.1).

## Results

### Establishment of provincial network to improve the ‘1-3-7’ approach

**Fig. 1:**
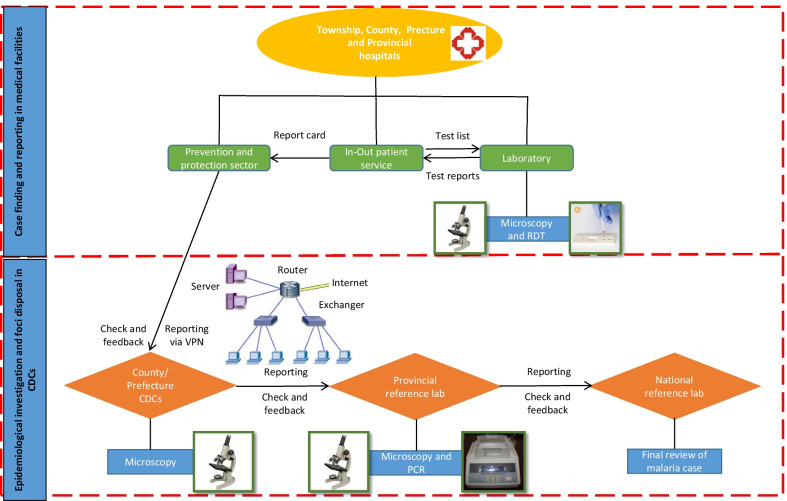
Malaria surveillance and response network in Jiangsu Province, China. *CDC* Center for Diseases Control and Prevention; *RDT* Rapid Diagnostic Tests; *VPN* Virtual private network

Jiangsu Province has established a malaria surveillance and response system network for malaria case finding, reporting via CRDSIS within one day, epidemiological investigation within three days, and focal/foci disposition within seven days. The system network covers provincial, prefectural, county and township levels for medical facilities and health providers (Fig. 1). In 2012, the Jiangsu provincial malaria diagnosis reference laboratory was established. The reference laboratory confirms *Plasmodium* species designations of all reported and suspected malaria cases and issues a monthly feedback report. In Jiangsu Province, medical facilities above the county level carry out microscopy using blood smears of malaria cases. The CDC personnel in different administrative levels are responsible for the functioning of the ‘1-3-7’ approach and the steps involved in the network system.

### Population health education to improve healthcare seeking behavior

Following the guidance of the Health Administrative Department in combination with annual publicity activities of National Malaria Day on April 26, local CDC staffs conducted population-level health education campaigns on malaria by designing slogans, displaying posters, broadcasting audio-visual materials, and distributing leaflets and traditional informational pamphlets to residents and high-risk populations such as laborers engaged in outdoor work including as infrastructure construction, lumberjacks, and truck drivers in African and Southeast Asian countries. Simultaneously, and jointly with multi-sector cooperation, malaria control and prevention knowledge was disseminated through newspapers, radio, television, and other multimedia outlets. In recent years, popular social media platforms such as *WeChat* and *Weibo* have been widely used to disseminate malaria related knowledge.

Throughout the implementation of ‘1-3-7’ approach from 2012 to 2020, an estimated 61.57 million individuals have benefited in Jiangsu Province. During the annual National Malaria Week in April, public health education campaigns were carried out including: 10,483 educational DVDs distributed, 9,446 radio/television ads displayed on knowledge of malaria control, 2,548 malaria control-related articles were published in newspapers, 23,745 pieces of exhibition boards were displayed, and 49,994 bulletin boards were posted. A total of 8,023,732 leaflets were printed and 1,201,076 brochures were distributed to residents. In-person educational opportunities included 28,172 malaria knowledge consultation sessions by CDC personnel and 6,384 malaria training courses were held for medical facilities’ staff and CDC’s personnel which benefited 61.57 million people across whole province (Table 1) during the 2012–2020 period. Since 2012, the Jiangsu Institute of Parasitic Diseases (JIPD) has designed and distributed 27 types of publicity materials, including leaflets, posters, and mugs with printed malaria prevention and control information for potentially high-risk populations and local residents.

**Table 1:** Publicity activities on annual National Malaria Day in Jiangsu Province, 2012–2020

Year	Publicity activity type									Number of beneficiaries (Population)
	Video views (times)	Radio/TV (sessions)	Newspaper (column)	Exhibition board (piece)	Bulletin board (piece)	Printed leaflets (*n*)	Brochures (*n*)	Consultation of malaria prevention knowledge (*n*)	Training course (*n*)	
2012	457	1,107	812	2,635	11,048	1,410,304	113,778	3,050	600	16,846,407
2013	1,023	1,586	619	2,801	10,126	1,188,993	135,458	3,579	616	7,825,118
2014	910	1,315	133	2,542	6,240	785,751	77,591	2,400	1,052	6,417,112
2015	761	1,054	128	2,020	3,994	652,274	121,004	3,350	771	5,952,338
2016	785	936	141	2,794	3,017	832,253	144,934	3,857	750	5,063,556
2017	1,323	787	93	2,587	3,453	818,988	198,926	3,038	631	6,010,751
2018	2,201	806	219	2,817	4,146	1,045,964	176,059	3,473	754	4,760,355
2019	1,266	750	180	3,126	3,639	765,213	142,805	2,995	716	4,423,164
2020	1,757	1,105	223	2,423	4,331	523,992	90,521	2,430	494	4,270,917
Total	10,483	9,446	2,548	23,745	49,994	8,023,732	1,201,076	28,172	6,384	61,569,718

For the consultation of malaria prevention knowledge, local CDC’s staffs have conducted. For the malaria training course, local medical facilities’ personnel and CDC’s staffs attended

### Time of healthcare seeking and malaria diagnosis of imported cases

Among the 2,423 imported malaria cases during 2012–2020, the mean and inter-quartile range of days elapsing between symptom onset and initial healthcare seeking was 1.29 days (range 0, 3). For malaria cases seeking healthcare services, 687 (28.4%) and 1,104 (45.6%) cases visited hospitals on day zero and two after symptom onset, respectively. Of the 2,423 cases, 632 visited hospitals more than three days after symptom onset (632/2,423; 26.1%). During the nine-year period, 55 (27.8%), 82 (24.1%), 88 (24.8%), 136 (33.6%), 63 (20.5%), 72 (30.1%), 83 (34.1%), 76 (31.1%) and 31 (34.4%) patients were diagnosed on day zero after seeking healthcare, respectively. For malaria cases seeking healthcare services on the same day of symptom onset, significant differences were identified during the nine year study period (*χ*^*2*^ = 27.27, *P* = 0.001) (Table 2).

**Table 2:** Time of healthcare seeking and malaria diagnosis of imported cases in Jiangsu Province, 2012–2020

Year	Cases seeking healthcare after symptom onset, *n* (%)			Diagnosis by medical facilities after symptom onset, *n* (%)			Total
	Day zero to seek healthcare	1–2 days to seek healthcare	≥ 3 days to seek healthcare	Day zero for diagnosis	1–2 days for diagnosis	≥ 3 days for diagnosis	
2012	55 (27.8)	67 (33.8)	76 (38.4)	106 (53.5)	40 (20.2)	52 (26.3)	198 (100.0)
2013	82 (24.1)	164 (48.1)	95 (27.8)	230 (67.5)	55 (16.1)	56 (16.4)	341 (100.0)
2014	88 (24.8)	168 (47.4)	99 (27.8)	227 (63.9)	66 (18.6)	62 (17.5)	355 (100.0)
2015	136 (33.6)	181 (44.7)	88 (21.7)	271 (66.9)	85 (20.9)	49 (12.2)	405 (100.0)
2016	63 (20.5)	168 (54.5)	77 (25.0)	201 (65.3)	67 (21.8)	40 (12.9)	308 (100.0)
2017	72 (30.1)	97 (40.6)	70 (29.3)	139 (58.2)	63 (26.3)	37 (15.5)	239 (100.0)
2018	83 (34.1)	106 (43.7)	54 (22.2)	132 (54.3)	68 (27.9)	43 (17.8)	243 (100.0)
2019	76 (31.1)	114 (46.8)	54 (22.1)	149 (61.1)	69 (28.3)	26 (10.6)	244 (100.0)
2020	31 (34.4)	39 (43.3)	20 (22.3)	47 (52.2)	27 (30.0)	16 (17.8)	90 (100.0)
Total	687 (28.4)	1,104 (45.6)	632 (26.1)	1,502 (61.9)	540 (22.3)	381 (15.7)	2,423 (100.0)

### Malaria diagnosis in different medical facilities

According to the surveillance and response procedures and ‘1-3-7’ approach in Jiangsu Province, malaria trainings on diagnosis and treatment, and rapid diagnostic test (RDT) usage for health facilities strengthened during the study period. Between 2012 and 2020, 2,423 imported malaria cases were reported via CRIDIS. Of the total, 1,820 (75.1%) malaria patients were diagnosed in all levels of medical facilities, and 562 (23.2%) cases were diagnosed in CDCs, including 41 (1.7%) cases diagnosed in China’s Entry-Exit Inspection and Quarantine Bureau (CIQ, China Customs). No malaria cases were diagnosed by village clinics or private clinics (Table 3). The mean and inter-quartile range of days between initially seeking healthcare and malaria diagnosis was 2.13 days (range: 0–3) during the study period. For diagnosis the same day or within two days of symptom onset by medical facilities, 1,502 (61.9%) and 540 (22.3%) cases were identified, respectively. Of the 2,423 imported cases, only 381 (15.7%) were diagnosed at three days or later. Between 2012 to 2020, a total of 1,502 (61.9%) patients were diagnosed on day zero after symptom onset, respectively. For the percentage of malaria cases confirmed on the same day as symptom onset, significant differences were observed between years (*χ*^*2*^ = 27.75, *P* = 0.001). For malaria diagnosis in the CDCs, the proportion of malaria cases diagnosed by CDCs professional personnel decreased over the year (*χ*^*2*^ = 171.35, *P* < 0.001). On the contrary, the proportion of malaria cases diagnosed by all levels of hospitals increased yearly (*χ*^*2*^ = 144.93, *P* < 0.001) (Table 3).

**Table 3:** Malaria diagnosis in different medical facilities in Jiangsu Province, 2012–2020

Diagnosis units	Years									
	2012 *n* (%)	2013 *n* (%)	2014 *n* (%)	2015 *n* (%)	2016 *n* (%)	2017 *n* (%)	2018 *n* (%)	2019 *n* (%)	2020 *n* (%)	Total
Provincial CDCs	0 (0.0)	1 (0.3)	0 (0.0)	0 (0.0)	1 (0.3)	0 (0.0)	0 (0.0)	0 (0.0)	0 (0.00)	2 (0.1)
Provincial medical facilities	8 (4.0)	9 (2.6)	5 (1.4)	6 (1.5)	4 (1.3)	4 (1.7)	5 (2.1)	5 (2.0	1 (1.1)	47 (1.9)
Prefectural CDCs	22 (11.1)	11 (3.2)	17 (4.8)	7 (1.7)	7 (2.3)	4 (1.7)	2 (0.8)	0 (0.0)	0 (0.0)	70 (2.9)
Prefectural medical facilities	59 (29.8)	76 (22.3)	106 (29.9)	158 (39.0)	127 (41.2)	113 (47.2)	99 (40.7)	123 (50.4)	40 (44.5)	901 (37.2)
County CDCs	53 (26.9)	121 (35.5)	100 (28.2)	74 (18.3)	60 (19.5)	30 (12.6)	32 (13.2)	16 (6.6)	4 (4.4)	490 (20.2)
County medical facilities	47 (23.7)	106 (31.1)	108 (30.4)	141 (34.8)	94 (30.5)	73 (30.5)	88 (36.2)	89 (36.5)	40 (44.5)	786 (32.4)
Township hospital	5 (2.5)	11 (3.2)	9 (2.5)	17 (4.2)	8 (2.6)	10 (4.2)	14 (5.8)	8 (3.3)	4 (4.4)	86 (3.6)
Nanjing Customs	4 (2.0)	6 (1.8)	10 (2.8)	2 (0.50)	7 (2.3)	5 (2.1)	3 (1.2)	3 (1.2)	1 (1.1)	41 (1.7)
Total	198 (100.0)	341 (100.0)	355 (100.0)	405 (100.0)	308 (100.0)	239 (100.0)	243 (100.0)	244 (100.0)	90 (100.0)	2,423 (100.0)

Zero locally indigenous cases was reported from 2012 to 2020 and all malaria cases were imported overseas

### Indicators of the ‘1-3-7’ approach

In order to improve the implementation quality of ‘1-3-7’ approach, tabletop exercises were conducted on malaria foci disposal to evaluate the performance of CDC personnel and local medical facility staff related to the ‘1-3-7’ approach indicators. Since 2012, 100.0% of malaria cases were reported via CRDSIS by local medical facility staff within one day (24 h) of case presentation. More than 99.4% of malaria cases received individual epidemiological investigations by local CDC staff at the county level within three days and more than 98.3% were disposed of within seven days (Table 4). The proportion of foci investigated within seven days decreased to 90.0% in 2020.

**Table 4:** Indicators of the ‘1-3-7’ approach in Jiangsu Province, 2012–2020

Year	Case detection and notification			Case investigation		Focus investigation and action	
	Number of imported malaria cases	Number of cases reported within 1 day	Proportion of cases reported within 1 day (%)	Number of cases investigated within 3 days	Proportion of cases investigated within three days (%)	Number of foci investigations and action taken within seven days	Proportion of foci investigations and action taken within 7 days (%)
2012	198	198	100.0	193	98.9	191	96.5
2013	341	341	100.0	341	100.0	341	100.0
2014	355	355	100.0	355	100.0	355	100.0
2015	405	405	100.0	405	100.0	405	100.0
2016	308	308	100.0	308	100.0	308	100.0
2017	239	239	100.0	231	97.9	223	93.3
2018	243	243	100.0	242	99.6	240	98.7
2019	244	244	100.0	244	100.0	238	97.5
2020	90	90	100.0	90	100.0	81	90.0
Total	2,423	2,423	100.0	2,409	99.4	2,382	98.3

### Progress of malaria elimination in Jiangsu Province

**Fig. 2:**
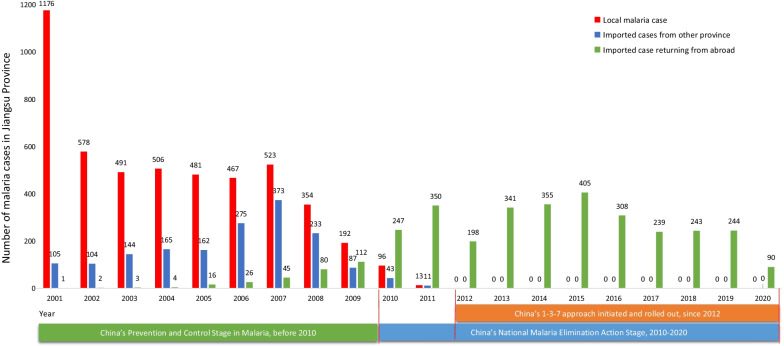
The changing malaria situation during 2001–2020 in Jiangsu Province, China

From 2001 to 2020, a total of 4,877 indigenous and 5,002 imported malaria cases were reported in Jiangsu Province (Fig. 2). The majority of malaria reported between 2001 to 2010 were indigenous cases (69.0%, or 4,864 cases); 1,615 cases (22.9%) were imported from other provinces within China, and 568 cases (8.1%) were imported from other countries. From 2011 to 2020, the total of 2,797 malaria cases were reported. Of them, there were 2,784 cases were imported from other countries and only 13 of them were due to indigenous *Plasmodium vivax* in Jiangsu Province (all in 2011). In 2012, indigenous vivax malaria cases were eliminated, and since then all malaria cases in Jiangsu Province were imported from abroad. In the recent decade, there have been 91 severe malaria cases and eight malaria deaths, all of which were imported. There have been no introduced malaria cases in Jiangsu Province during the study period.

**Fig. 3:**
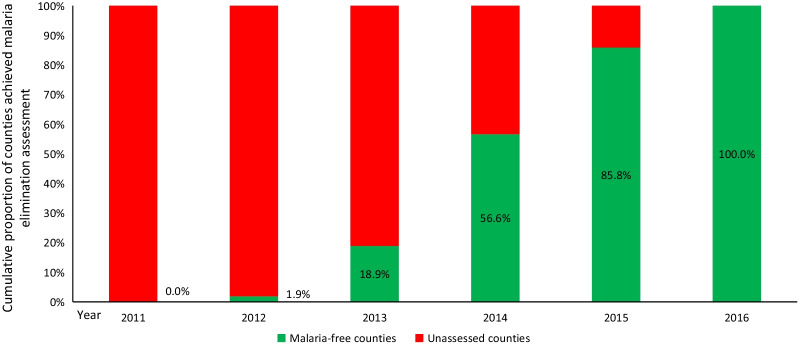
Malaria elimination progress from 2011 to 2020 in Jiangsu Province, China

Beginning in 2012, Jiangsu Province initiated a county-level assessment strategy of malaria elimination progress. All 100 counties in Jiangsu Province were individually monitored and assessed on their progress toward malaria elimination, of which, Sheyang county and Liyang county first achieved the criteria for elimination and passed the assessment. By the end of 2016, all 100 counties in Jiangsu Province passed the county-level assessment. In 2017, all 13 prefectural cites in Jiangsu Province passed the municipal-level assessment of malaria elimination, and the whole province achieved the goal of malaria elimination which has since been maintained since (Fig. 3).

## Discussion

Since the NMEAP was initiated in China in 2010, the ‘1-3-7’ approach was developed and applied in Jiangsu Province before soon being scaled up to all 31 PLADs of China in 2012. Jiangsu Province reported its last indigenous malaria case in 2011, and interrupted local malaria transmission by 2012. In 2018, the provincial level malaria elimination assessment organized by NHC was conducted in Jiangsu and the province was certified as malaria-free. Jiangsu Province documented important experiences in controlling and eliminating malaria, including preventing the re-introduction of malaria as counties within Jiangsu Province began experiencing greater imported malaria. Local CDC staffs of Jiangsu Province has conducted population-level health education to improve healthcare-seeking behavior, strengthened capacity of health facility personnel to improve the performance of malaria diagnosis and treatment, and of public health providers to improve implementation of the ‘1-3-7’ approach. Jiangsu Provincial Malaria Weekly [16], first issued in September 2011, was a weekly newsletter aimed to strengthen the implementation of the ‘1-3-7’ approach by sharing information on activities being conducted at the county-level. The newsletter includes information on malaria endemic trends, key indicators related to the ‘1-3-7’ approach, information to provide patients who went abroad and will return. The Jiangsu Provincial Malaria Weekly delivered by mail and urged all levels of CDC staffs to comply with the requirements outlined in the malaria elimination guidelines. The innovative Jiangsu Provincial Malaria Weekly emphasized that in achieving and sustaining malaria elimination, the surveillance and response system, and those involved in the system, should be vigilant and effective in their response to remove any local malaria transmission.

The malaria surveillance and response network in Jiangsu Province played a key role in the progress of malaria elimination. Malaria cases in Jiangsu Province are diagnosed at different medical facility levels within the network. The proportion of malaria cases diagnosed by local CDCs decreased over time as more malaria patients sought care in hospitals. The increased awareness of early healthcare seeking of malaria patients and diagnostic capacity of medical staffs at different medical facility levels, including hospitals, largely contributed to the health education efforts on malaria for both medical staff and higher-risk groups for malaria. For example, migrant workers or business people who travel to malaria-endemic areas abroad were able to have access to a medical facility that is trained and aware of the signs and symptoms of malaria. Typically, local CDC staff are mainly responsible for blood smear microscopy, case confirmation, individual case epidemiological investigation and foci disposition. The Provincial malaria reference laboratory are responsible for checking and confirming the positive or negative results of blood samples reported from local CDC and township hospitals (community health centers), so as to ensure quality of blood test for suspected patients such as in Jiangsu Province.

Timely treatment-seeking behaviors for healthcare services by malaria patients is an important way to identify malaria cases [17]. Most imported malaria cases visited a hospital (73.9%) seeking healthcare services after experiencing fever, chills or sweating within two days of onset. However, only 26.1% of patients during the study period didn’t visit a medical facility or seek healthcare advice for at least three days or more. The findings suggest that patient treatment-seeking delays still exist, possibly due to low malaria knowledge and higher medical expenses [18]. Most of the patients in Jiangsu Province, and also within China, are laborers engaged in outdoor work such as infrastructure construction, lumberjacks, or truck drivers in African and Southeast Asian countries [19]. Therefore, their protective measures and self-protection awareness are relatively poor. Moreover, their awareness for attending health facilities for malaria treatment are usually much lower compared with those who are not traveling for occupational reasons [20].

Public health activities including the mass distribution of educational materials on National Malaria Day played an important role in the process of eliminating malaria. This study identified a variety of key formats and channels for health education intervention distribution on malaria. In the past decade, local CDC, community health centers, and CIQ staff has carried out health education interventions using different kinds of publicity activities in villages and communities where returning laborers reside. This played an important role in increasing the knowledge and awareness of high-risk populations who traveled to highly malarious areas such as sub-Saharan Africa and Southeast Asia [21]. In recent years, newly developed health education channels, such as *WeChat*, have been used. These types of medias have been proven to be an effective, sustainable, feasible, and well-accepted strategies for improving health [22]. Due to the negative impact of the global COVID-19 pandemic [23], using social media channels to supply information about the prevention and treatment of malaria through smartphones has been an effective and safe method to target high-risk populations, for example, in travelers returning from higher-burden malaria areas to China.

Most imported cases were diagnosed within the prescribed timeframe, with less than 16.7% of patients the who were not diagnosed within three days of symptom onset. If suspected malaria patients have persistent fever, local medical doctors should be vigilant to inquire about recent travel history. Furthermore, village doctors and township health workers should provide a timely referral of suspected malaria patients to county or city health facilities [24]. Delays in health-seeking behavior, diagnosis, and/or treatment for malaria are associated with an increased risk for severe disease and mortality [17]. There is a need to further improve the health care-seeking behavior of patients, as well as the diagnostic capacity of the health care facilities they visit. An insufficient capacity for malaria diagnosis has been observed in Jiangsu Province [25], especially in the preparation and reading of blood smears at the township and village levels. RDT implementation in Jiangsu Province has improved access to healthcare among malaria patients in rural areas and villages, and could reduce delayed diagnosis of malaria patients [26, 27].

Regarding the functioning of the ‘1-3-7’ approach in Jiangsu Province, a total of 100% of cases were reported within one day, 99.4% of cases were investigated within three days, and 98.3% of foci responses and disposition were achieved within seven days. The ‘1-3-7’ approach has been implemented nationwide since early 2012, and has been a key strategy in detecting, treating and responding to individual malaria cases and eliminating the source of infection in a regulated and timely fashion. Importantly, the ‘1-3-7’ approach was not only adopted as guidance in the *Malaria surveillance, monitoring & evaluation: A Reference Manual* [10] by the World Health Organization, but has been adapted as a surveillance and working model by many other countries such as Cambodia [28] Thailand [29] and Tanzania [30], among others.

To improve the functioning of the ‘1-3-7’ approach, important experiences and lessons from Jiangsu can be shared, adapted, and adopted in other countries for local context. Firstly, monitoring and evaluation of malaria case investigation in malaria-eliminating settings such as Jiangsu Province [31] can also improve programme performance to the high level that is required to reach elimination. Secondly, to maintain the diagnostic capacity in an elimination setting, provincial level parasitic diagnostic skills competitions [32] were organized regularly and were critically important. In doing so, Jiangsu Province was able to maintain and improve the competency of microscopy examiners for local medical facilities and provide strong technical support for malaria elimination. Local hospitals can improve capacity of clinical diagnosis and treatment of imported malaria and reduce incidence of severe cases and death cases. More interactive and discussion-based methods, such as the tabletop exercises, could be used to maintain the malaria specific skills of medical facility staff that are at the frontlines of healthcare in local counties and villages. This strategy could also increase the knowledge of malaria-specific personnel on China’s ‘1-3-7’ approach in city, county and township level CDCs [33]. The Chinese ‘1-3-7’ approach has proven to be successful, but can always be improved [34].

This study summarized the measures and experiences through a retrospective analysis of imported malaria cases into China, which could be adapted and applied in other countries seeking for malaria free. One limitation of this study is that the working mechanism and measures for malaria prevention and control in multi-sector cooperation such as the customs departments was not introduced for detail due to the limited data acquisition.

## Conclusions

Jiangsu Province has established an efficient and sensitive system for surveillance and response to achieve malaria elimination and successfully manage imported malaria cases returning from abroad. The improved surveillance and response system plays an important role in high-risk areas susceptible to importation or increased transmission, as well as during potential malaria-related outbreak emergencies in China. Maintaining key malaria knowledge for suspected patients and their treatment-seeking behaviors, timely and accurate malaria diagnosis competency among health staff, and ensuring real-time reporting of cases, and rapid foci disposition capacity by local malaria personnel remains a critical focus for China to sustain its malaria freedom.

## Data Availability

The data will be available upon requested.

## Abbreviations

NHC: National Health Commission
NMEAP: National Malaria Elimination Action Plan
CDCs: Centers for Diseases Control and Prevention
WHO: World Health Organization
CRDSIS: China’s Routine Diseases Surveillance Information System
RDT: Rapid Diagnostic Tests
CIQ: China’s Entry-Exit Inspection and Quarantine Bureau
